# Outcomes of endovascular treatment versus bypass surgery for critical limb ischemia in patients with thromboangiitis obliterans

**DOI:** 10.1371/journal.pone.0205305

**Published:** 2018-10-09

**Authors:** Chung Yeop Lee, Kyunghak Choi, Hyunwook Kwon, Gi-Young Ko, Youngjin Han, Tae-Won Kwon, Yong-Pil Cho

**Affiliations:** 1 Department of Surgery, University of Ulsan College of Medicine and Asan Medical Center, Seoul, Republic of Korea; 2 Department of Radiology, University of Ulsan College of Medicine and Asan Medical Center, Seoul, Republic of Korea; University of Massachusetts Medical School, UNITED STATES

## Abstract

We aimed to compare the clinical outcomes between endovascular treatment and inframalleolar bypass surgery for critical limb ischemia (CLI) in patients with thromboangiitis obliterans (TAO) and to assess the role of bypass surgery in the era of innovative endovascular treatment. Between January 2007 and December 2017, a total of 33 consecutive patients with the diagnosis of TAO presenting with CLI who underwent endovascular treatment (endovascular group, n = 22) or bypass surgery to the pedal or plantar vessels (bypass group, n = 11) were included and analyzed retrospectively. The primary endpoint was defined as a major amputation of the index limb, and the secondary endpoint was defined as graft occlusion, regardless of the number of subsequent procedures. In the bypass group, six patients (55%) had undergone previous failed endovascular procedures and/or arterial bypass surgery to the index limb before inframalleolar bypass, and two patients (18%) received microvascular flap reconstruction after bypass surgery. During the median follow-up period of 32 months (range 1–115 months), there were no significant differences in primary and secondary endpoints between the two groups although the bypass group had a higher Rutherford class than the endovascular group. Kaplan–Meier survival analysis showed that there were similar limb salvage (*P* = 0.95) and graft patency rates (*P* = 0.39). In conclusion, endovascular treatment is a valid strategy leading to an acceptable limb salvage rate for TAO patients, and surgical bypass to distal target vessels could play a vital role in cases of previous failed endovascular treatment or extensive soft tissue loss of the foot.

## Introduction

Although arterial bypass with autogenous vein is regarded as the ideal management strategy for patients with severe atherosclerosis obliterans (ASO) affecting the crural arteries presenting with symptoms and signs of critical limb ischemia (CLI), it is not always feasible to perform in patients with thromboangiitis obliterans (TAO). TAO frequently involves the small distal arteries of the calf and the foot causing complete obliteration of the vessel lumen, and superficial veins, used as vascular conduits for bypass surgery, may become affected by the disease in the form of phlebitis migrans with consecutive inflammatory changes [1–3]. During the past two decades, the less invasive endovascular procedure has become an alternative approach for the management of ASO due to the advances in devices and has also been attempted more frequently in TAO patients [1]. However, unsuccessful endovascular treatment or repeated occlusions after an endovascular procedure are indications for bypass to the pedal or plantar arteries in selected patients. Furthermore, even after successful endovascular treatment, patients with extensive soft tissue loss of the foot may not heal spontaneously, requiring microvascular free flap transfers to cover large defects and gangrenous areas for limb salvage; these patients might benefit from surgical bypass of the foot arteries [4–7]. For patients with CLI with no option for endovascular or surgical treatment, distal vein bypass (venous arterialization), using the disease-free venous bed as an alternative conduit for perfusion of the peripheral tissues with arterial blood, could be a valuable treatment option for limb salvage, with a reported limb salvage rate of 75% at 12 months [8,9].

The aims of the study were to compare the clinical outcomes between endovascular treatment and bypass surgery to the foot arteries or veins in TAO patients presenting with CLI and to assess the role of distal bypass surgery in the era of innovative endovascular treatment.

## Materials and methods

### Study design and population

This single-center, retrospective, observational study was based on data from a prospectively maintained registry. The study protocol was approved by the Institutional Review Board of Asan Medical Center, which waived the need for informed consent. Between January 2007 and December 2017, of the 177 consecutive patients with the diagnosis of TAO, 33 patients (18.6%) presenting with CLI (Rutherford class 4–6) who underwent endovascular treatment or bypass surgery to the pedal or plantar arteries or veins were included in the study [10,11]. The diagnosis of TAO is based on Shionoya’s criteria and arteriographic findings [12].

Our management strategy for TAO is determined based on symptoms and signs of limb ischemia (Rutherford class) [10]; conservative treatment including cessation of smoking, possible use of antiplatelet or vasodilator therapy, and local wound care, is recommended as the first line treatment in patients without CLI (Rutherford class 1–3) whereas endovascular treatment with conservative treatment is recommended in selected patients with CLI or whose local symptoms do not improve after conservative treatment. Bypass surgery is strictly indicated for CLI patients with long occlusions in jeopardy of losing a foot or leg, or when a previous endovascular procedure fails. In our analysis, endovascular treatment in patients without CLI was excluded.

The demographics and other data for all consecutive patients, including clinical and anatomical characteristics, procedural details, postoperative outcomes, and follow up data were recorded prospectively in an Excel database (Microsoft Corp., Redmond, WA, USA) or extracted retrospectively from the patients’ case records.

### Preoperative assessments and details of the procedure

All patients underwent ankle-brachial index assessment with or without toe-brachial index to evaluate hemodynamic parameters and spontaneous healing potential, and computed tomography angiography (CTA) and/or conventional digital subtraction angiography (DSA) to delineate the features of the arterial lesions. All patients were given general recommendations about complete smoking cessation.

All endovascular procedures were performed under local anesthesia as previously published [1]. For all bypass surgery procedures, the suitability of the inflow and outflow arterial anatomy was initially evaluated by means of CTA and/or DSA. If there was no visible recipient artery on the CTA or DSA, duplex ultrasound (DUS) was performed to find a potentially open recipient artery or vein in the foot. Autogenous vein grafts were used as a vascular conduit whenever possible. Before the bypass surgery procedures, the autogenous veins were evaluated with DUS. The bypass surgery was performed under general anesthesia. All autogenous venous grafts were great saphenous veins (GSVs), but small saphenous veins or arm veins were also harvested when the GSV was not adequate or not long enough for bypass surgery. If a single segment vein graft was not available, a spliced vein graft with two or more segments was applied. In cases where sufficient length of the autogenous venous graft was not obtained, prosthetic-vein composite grafts were used as an alternative vascular conduit for bypass surgery. The harvested GSVs or other autogenous venous grafts were used in a non-reversed configuration after disrupting the valves for graft-to-artery or vein size-matching and bidirectional flow to reduce stasis in the graft. Unfractionated heparin was administered intraoperatively as a bolus of 5000 IU. The distal anastomosis was reconstructed in an end-to-side fashion. Operative adjuncts for distal anastomosis such as a Linton patch [13] and arteriovenous fistula creation were used in cases with poor distal runoff artery without obliteration, whereas distal vein bypass of the foot was performed in cases with an obliterated distal runoff artery. Completion angiography was carried out to ensure adequate graft flow at the end of the operation.

### Postoperative care and surveillance

According to our protocol, anticoagulation with intravenous heparin followed by oral warfarin therapy in combination with dual antiplatelet therapy was administered for 12 months after the procedure. Thereafter, single antiplatelet therapy was prescribed indefinitely. Our routine surveillance program included follow up visits within 1 month of the procedure, 3 and 6 months after the procedure, and every 6 months thereafter to monitor limb status as well as graft patency. Patients with open wounds or with a secondary procedure were followed up more frequently. DUS of the entire graft and both anastomoses with or without CTA was performed within 1 month of the procedure, 6 and 12 months after the procedure, and annually thereafter if the evaluations showed no abnormalities.

### Definitions and study endpoints

Amputation was classified as either major (above the ankle) or minor (below the ankle) [1]. The graft patency was assessed using imaging studies (DUS or CTA); primary patency was defined as the interval from the initial procedure until the repeat procedure or a major amputation of the index limb, whichever occurred first, and secondary patency was defined as the interval from the initial procedure until the graft occlusion or a major amputation of the index limb, regardless of the number of subsequent procedures. The primary endpoint was a major amputation of the index limb during the study period, and the secondary endpoint was graft occlusion, regardless of the number of subsequent procedures.

### Statistical analysis

Categorical variables are reported as frequencies and percentages, and continuous variables as medians and ranges. Categorical variables were compared using the chi-squared test, or Fisher’s exact test when the chi-squared test was not available, whereas continuous variables were compared using Student’s *t-*test. In one patient lost to follow-up in the bypass group, the last available outcome was retained in the analysis. A Cox proportional hazards model was used to identify possible risk factors associated with primary (major amputation) and secondary (graft occlusion) endpoints in the whole study group. Survival curves were constructed using Kaplan–Meier estimates and were compared using the log-rank test. *P* <0.05 was considered statistically significant. Statistical analyses were performed with SPSS Version 21.0 (SPSS Inc., Chicago, IL, USA).

## Results and discussion

Of the 177 consecutive patients with the diagnosis of TAO, 33 patients presenting with CLI who underwent an endovascular procedure (n = 22, 67%) or bypass surgery (n = 11, 33%) were included in the analysis; we excluded 144 patients not indicated for endovascular or surgical treatment. Among the 144 patients excluded from this study, 5 patients (3.5%) underwent major amputation without any interventions (3 patients) or failed endovascular treatment at outside facility (2 patients). The baseline and clinical characteristics of the study population are presented in Table 1. The median age was 42 years (range, 20–71 years), and 97% of the patients were male. All patients were current (n = 25, 76%) or past (n = 8, 24%) smokers but were without serious cardiopulmonary comorbidities. There were no significant differences between the endovascular and bypass groups in demographics, smoking history, and arterial lesion involvement, except that patients who had bypass surgery were younger (*P* = 0.03) and had a higher Rutherford class (*P* = 0.01) than those who had an endovascular procedure. Endovascular procedures were performed as follows: plain balloon angioplasty in 12 patients, stent placement in 6 patients, and thrombolysis with plain balloon angioplasty in 4 patients. In the bypass group, the indication for bypass surgery was an ulcer or gangrene (Rutherford class 5 or 6), but 45% of the patients had rest pain alone (Rutherford class 4) in the endovascular group. Operative details for the bypass group are given in Table 2. Six patients (55%) had undergone previous failed endovascular procedures and/or arterial bypass surgery to the index limb before inframalleolar bypass, and three (27%) had previously received other procedures such as sympathectomy and minor amputation. Distal vein bypass was performed in three patients (27%) and operative adjuncts were done in eight patients (73%).

**Table 1:** Baseline and clinical characteristics of the study population.

	Total (n = 33)	Endovascular group (n = 22)	Bypass group (n = 11)	P-value
Median age, years	42 (20–71)	46 (20–71)	41 (22–52)	0.03
Male sex	32 (97)	21 (95)	11 (100)	>0.99
BMI, kg/m^2^	23.3 (14.5–32.8)	23.9 (14.5–32.8)	21.9 (19.9–31.7)	0.96
Smoking				
Current smoking	25 (76)	18 (82)	7 (64)	0.39
Past smoking	8 (24)	4 (18)	4 (36)	0.39
Pack-year	18 (2–100)	20 (5–100)	10 (2–45)	0.09
Rutherford class				
Class 4 (rest pain)	10 (30)	10 (45)	0	0.01
Class 5 (ulcer)	8 (24)	4 (18)	4 (36)	0.39
Class 6 (gangrene)	15 (45)	8 (36)	7 (64)	0.14
Class 5 & 6	23 (70)	12 (55)	11 (100)	0.01
Lesion involvement				
FP artery	4 (12)	4 (18)	0	0.28
TP artery	7 (21)	4 (18)	3 (27)	0.66
FP + TP artery	22 (67)	14 (64)	8 (73)	0.71

Continuous data are presented as medians (ranges); categorical data are given as numbers (%).

BMI, body mass index; FP, femoropopliteal; TP, tibioperoneal;

**Table 2:** Operative details of the bypass group.

Operative details	Number of patients (%)
Previous procedure	9 (82)
Endovascular procedure	2 (18)
Surgical procedure	2 (18)
Endovascular and Surgical procedure	2 (18)
Others	3 (27)
Inflow	
Femoral artery	4 (36)
Popliteal artery	7 (64)
Outflow	
Dorsalis pedis artery	3 (27)
Plantaris artery	5 (45)
Venous arterialization	3 (27)
Graft material	
Autogenous vein	8 (73)
Prosthetic-vein composite graft	3 (27)
Operative adjuncts	
Linton patch	4 (36)
Distal arteriovenous fistula	2 (18)
Microvascular flap reconstruction	2 (18)

The median follow-up was 32 months (mean 41 months, range 1–115 months). Although the bypass group had a higher Rutherford class than the endovascular group, there were no significant differences between both groups regarding primary and secondary endpoints. The primary endpoint was met in 9% of patients in both the endovascular and bypass groups (*P* >0.99); the secondary endpoint was met in 27% and 36%, respectively (*P* = 0.70) (Table 3). In the bypass group, three patients, not candidates for distal arterial revascularization due to the lack of a foot target artery, were treated by distal vein bypass and two patients underwent microvascular flap reconstruction to cover large defects and gangrenous areas of the foot after bypass surgery with preservation of a functional limb in all patients. At the end of the study period, all patients were alive; the primary patency rate was 55% in both groups (*P* >0.99), and minor amputation had been performed in 27% and 9%, respectively (*P* = 0.38).

**Table 3:** Clinical outcomes of the study population.

	Total (n = 33)	Endovascular group (n = 22)	Bypass group (n = 11)	P-value
Time interval (month)^a^	0 (0–214)	4 (0–252)	0 (0–214)	0.76
Follow-up (month)^b^	32 (1–115)	31 (1–113)	34 (1–115)	0.54
Primary endpoint	3 (9)	2 (9)	1 (9)	>0.99
Secondary endpoint	10 (30)	4 (18)	4 (36)	0.70
Minor amputation	7 (21)	6 (27)	1 (9)	0.38

Continuous data are presented as medians (ranges); categorical data are given as numbers (%).

^a^ Time interval between the diagnosis of TAO and procedure.

^b^ Follow up period after endovascular procedure or bypass surgery

**Fig 1:**
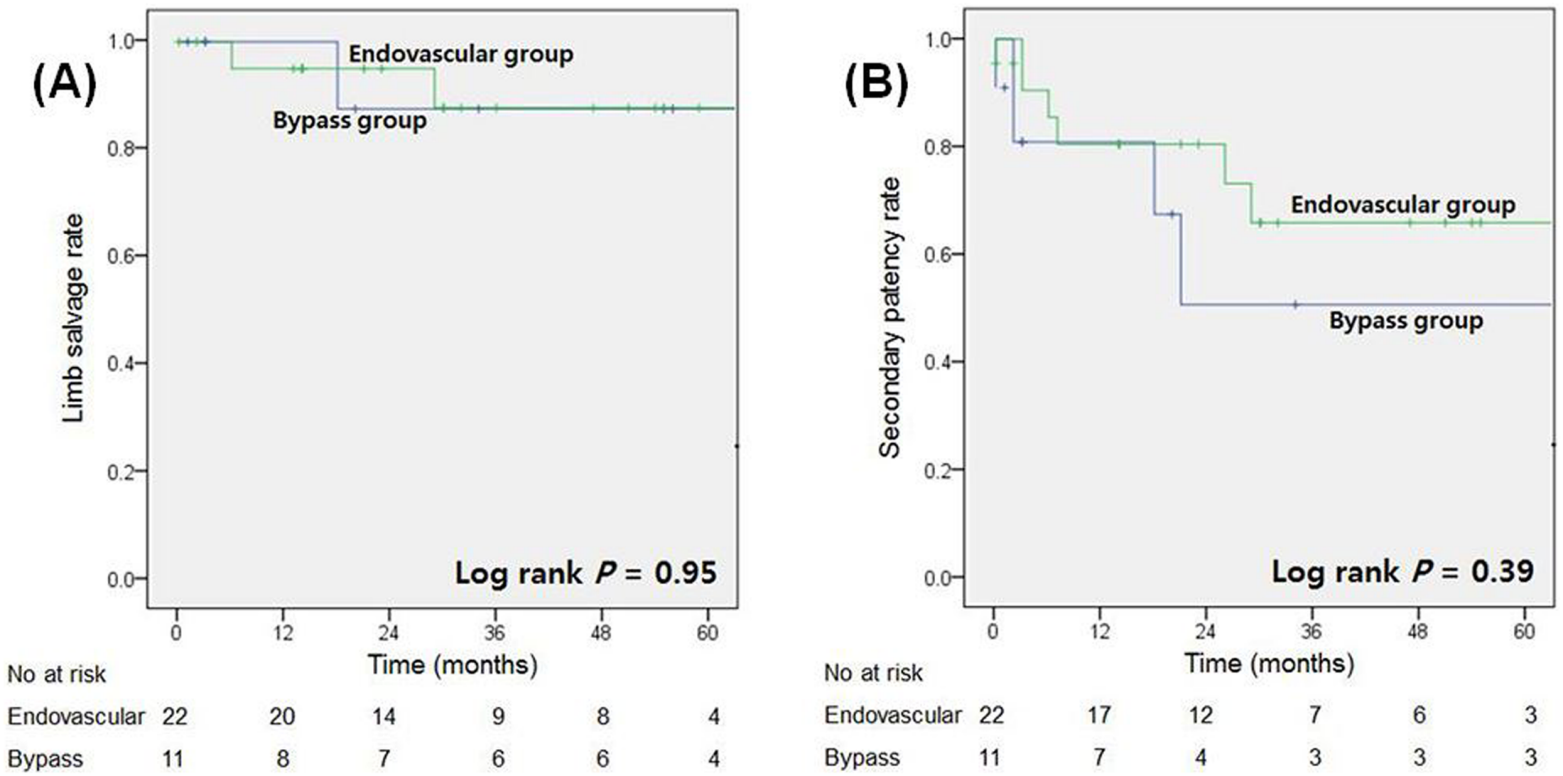
Kaplan–Meier survival analysis. Kaplan–Meier estimates of **(A)** limb salvage and **(B)** secondary patency rates of the index limb after endovascular procedure and bypass surgery.

Kaplan-Meier survival analysis showed that there were similar limb salvage (*P* = 0.95) and secondary graft patency rates (*P* = 0.39) in the two treatment groups (Fig 1). The respective limb salvage rates at 1 and 3 years were 96.4% and 87.4% in all included patients: 95.0% and 87.7% in the endovascular group, and 100% and 87.5% in the bypass group. Clinical primary and secondary patency rates were 58.4% and 49.0%, and 80.1% and 61.2% in all included patients at 1 and 3 years, respectively: 60.2% and 47.4%, and 80.4% and 65.8% in the endovascular group at 1 and 3 years, respectively: 53.9% and 53.9%, and 80.8% and 50.5% in the bypass group at 1 and 3 years, respectively. To investigate the independent clinical variables associated with limb salvage and graft patency, Cox proportional hazards regression analyses were performed, but there was no significant risk factor according to our analysis (S1 and S2 Tables).

TAO is regarded as a chronic inflammation of the small and medium-sized arteries and veins of both the lower and upper extremities with unknown etiology affecting younger smokers followed by a thrombotic occlusion [2,3]. Although medical treatment and smoking cessation are sometimes sufficient to induce a remission of the ischemic symptoms, resulting in relatively favorable outcomes for the affected limbs in contrast to patients with ASO, the resulting ischemia may lead to severe chronic symptoms such as non-healing ulceration and gangrene, and even major amputation [2,3]. In patients with TAO in jeopardy of losing a foot or leg, a more aggressive therapeutic option, either endovascular or surgical procedure, is required to not only avoid major amputation (allowing a return to ambulation) but also to stabilize the socioeconomic situation (allowing a return to work) of these relatively young patients [14]. It was reported that once healing of the lesions is achieved, the clinical situation may remain stable with preservation of a functional limb despite re-occlusion of the treated arteries [15,16]. Furthermore, particularly in some patients with large tissue defects, surgical treatment combining distal bypasses with microvascular free flap transfers to cover the local defect at the foot showed encouraging results [5–7].

Based on our review of the patient outcomes under our management strategy, we found that the results of endovascular treatment and distal bypass surgery were comparable, and no difference in the limb salvage and secondary graft patency rates was noted even though most inframalleolar bypass surgeries were carried out in cases with a higher Rutherford class or after a previous failed endovascular procedure and/or arterial bypass surgery. Furthermore, despite the small number of patients, the limb salvage results of distal vein bypass reported in the present study are encouraging in this group of patients for whom no other revascularization options were available and who probably would have undergone amputation if treated conservatively.

Endovascular treatment is a less invasive procedure and has shown promising technical success due to recent advances in devices [1]. Furthermore, endovascular treatment could be applied in patients without a distal runoff artery or without an adequate venous conduit for bypass surgery [1]. For TAO patients with CLI, endovascular treatment has resulted in excellent technical success as well as limb salvage rates according to several case series [17,18]. However, there was a high rate of re-intervention affecting the process of ulcer healing, and long-term results for these procedures are lacking in the literature. In contrast, excellent long-term results of distal artery bypass surgery have been reported as early as 20 years ago and successful arterial revascularization dramatically improves the symptoms of CLI and leads to an acceptable limb salvage rate for TAO patients [19–21]. Furthermore, distal artery bypass surgery can also be offered after failed or inadequate endovascular treatment; there are data suggesting that a prior endovascular procedure to treat crural occlusions does not have a negative impact on the outcome of a subsequent distal artery bypass as long as destruction of the target vessel is avoided and the bypass is performed without excessive delay [22]. Unfortunately, the arterial circulation of the lower calf is usually unavailable to serve as the distal anastomotic site or lacks a conduit for bypass surgery, so distal artery bypass surgery is not frequently performed in TAO patients [23,24]. Despite advances in endovascular and surgical techniques, a substantial number of TAO patients with CLI cannot be treated by any conventional therapeutic procedure and eventually require major amputation [8,9]. In our institution, distal vein bypass is often the last option for limb salvage in TAO patients with CLI when either endovascular treatment or distal artery bypass surgery is not available. Although controversy exists whether venous arterialization relieves CLI and prevents amputation [25,26]; theoretically, there are several reasons why venous arterialization might benefit patients [9]: (1) Reversal of flow all the way through the capillaries improves tissue nutrition; (2) flow in existing collateral vessels will increase; and (3) venous arterialization could stimulate angiogenesis. Recently published meta-analyses reported that venous arterialization may be considered as a viable alternative before major amputation is undertaken in patients with CLI and no other options, although the evidence was limited [8,9].

This study has several limitations. First, this was a retrospective analysis of single-center registry data from a relatively small number of patients in each of the two groups and thus was subject to selection and information biases. Therefore, the decisions to perform an endovascular or surgical procedure and whether to use operative adjuncts for distal anastomosis during bypass surgery were mainly made by the physician based on the expected level of technical difficulty of the procedure. The bypass group had a higher Rutherford class and the higher proportion of patients undergoing previous failed endovascular procedures and/or arterial bypass surgery to the index limb than the endovascular group because bypass surgery is more strictly indicated than endovascular treatment; operative wound of the foot in itself could be another significant risk factor of major amputation in the failed bypass surgery. Second, during the period of this study, the number of excluded patients was considerable because there is no consensus on the management of TAO patients with CLI except for smoking cessation. Third, we did not assess other important functional outcomes such as return to full ambulation and return to work, among others. Fourth, based on the small sample size, this study was likely underpowered to provide sufficient evidence supporting the overall relevance of our results. Finally, the follow-up duration may be insufficient to evaluate the long-term clinical outcomes in TAO patients with CLI receiving either an endovascular or surgical procedure. Future prospective trials with larger cohorts should lead to a better understanding of the efficacy of endovascular and surgical procedures in TAO patients with CLI and the role of inframalleolar bypass surgery in those with CLI and no other treatment options.

In conclusion, although the results of our small series must be regarded as preliminary given the limited follow-up, our findings indicate that endovascular treatment is a valid strategy leading to an acceptable limb salvage rate for selected TAO patients presenting with CLI who are refractory to conservative treatment. Despite a higher Rutherford class and the higher proportion of patients undergoing previous failed interventions, surgical bypass to distal target vessels could play a vital role for limb salvage in patients who are in jeopardy of losing a foot or leg.

## Supporting information

S1 Data Data of 33 consecutive patients with the diagnosis of TAO presenting with CLI (Rutherford class 4–6) who underwent endovascular treatment or bypass surgery to the pedal or plantar arteries or veins. (XLSX)

S1 Table Factors associated with primary endpoint (major amputation). (DOCX)

S2 Table Factors associated with secondary patency (graft occlusion). (DOCX)
