# Supplementary material for: Outcomes of endovascular treatment versus bypass surgery for critical limb ischemia in patients with thromboangiitis obliterans
Source: PLoS One. 2018 Oct 9;13(10):e0205305. doi: 10.1371/journal.pone.0205305 (PMC6177182; doi:10.1371/journal.pone.0205305)
Supplement: S1 Table — (DOCX) [file pone.0205305.s002.docx]

**S1 Table. Factors associated with primary endpoint (major amputation)**

|  | Univariate analysis |  |
| --- | --- | --- |
|  | HR (95% CI) | P-value |
| Age | 0.375 (0.034–4.149) | 0.424 |
| Male | 21.363 (0.000–) | 0.849 |
| Current smoker | 0.695 (0.063–7.671) | 0.767 |
| Rutherford class 4 | 1.093 (0.099–12.059) | 0.942 |
| Rutherford class 5 & 6 | 0.915 (0.083–10.092) | 0.942 |
| FP artery involvement | 27.172 (0.000–) | 0.626 |
| Endovascular treatment | 0.926 (0.084–10.246) | 0.950 |

CI, confidence interval; FP, femoropopliteal; HR, hazard ratio
