# Supplementary material for: Outcomes of endovascular treatment versus bypass surgery for critical limb ischemia in patients with thromboangiitis obliterans
Source: PLoS One. 2018 Oct 9;13(10):e0205305. doi: 10.1371/journal.pone.0205305 (PMC6177182; doi:10.1371/journal.pone.0205305)
Supplement: S2 Table — (DOCX) [file pone.0205305.s003.docx]

**S2 Table.** **Factors associated with secondary patency (graft occlusion)**

|  | Univariate analysis |  |
| --- | --- | --- |
|  | HR (95% CI) | P-value |
| Age | 0.660 (0.140–3.112) | 0.599 |
| Male | 21.480 (0.000–) | 0.699 |
| Current smoker | 0.484 (0.136–1.715) | 0.261 |
| Rutherford class 4 | 2.518 (0.727–8.713) | 0.145 |
| Rutherford class 5 & 6 | 0.397 (0.115–1.375) | 0.145 |
| FP artery involvement | 2.330 (0.294–18.470) | 0.423 |
| Endovascular treatment | 0.574 (0.161–2.050) | 0.393 |

CI, confidence interval; FP, femoropopliteal; HR, hazard ratio
